# Recurrent glomerulonephritis following renal transplantation and impact on graft survival

**DOI:** 10.1186/s12882-018-1135-7

**Published:** 2018-12-03

**Authors:** S. H. Jiang, A. L. Kennard, G. D. Walters

**Affiliations:** 1 Department of Renal Medicine, The Canberra Hospital, PO Box 11, Woden, Canberra, ACT 2605 Australia; 2 Department of Immunology and Infectious Diseases, John Curtin School of Medical Research, Australian National University, Canberra, Australia; 3 Australian National University Medical School, Canberra, Australia; 4 ANZDATA Registry, Adelaide, 5000 Australia

**Keywords:** Glomerulonephritis, Transplant, Disease recurrence

## Abstract

**Background:**

Recurrence of primary glomerulonephritis in the post-transplant period has been described in the literature but the risk remains poorly quantified and its impact on allograft outcomes and implications for subsequent transplants remain under-examined. Here we describe the rates and timing of post-transplant glomerulonephritis recurrence for IgA nephropathy, focal segmental glomerulosclerosis, mesangiocapillary GN and membranous GN based on 28 years of ANZDATA registry transplant data.

**Methods:**

We investigated the rates of GN recurrence and subsequent graft outcomes in 7236 patient from 28 years of ANZDATA transplant registry data. Data were analysed in R, using Kaplan Meier Survival analysis and adjusted analyses performed using Cox Proportional Hazards methods. A competing risk model was also analysed.

**Results:**

GN recurrence occurred in 10.5% of transplants and was most common in mesangiocapillary GN. Median time to recurrence was shorter for FSGS compared to IGAN. GN recurrence was less common in patients over 50 years of age and after unrelated kidney donation. We identified a significantly higher risk of recurrence in secondary grafts following recurrence in a primary allograft for FSGS (RR 5.70, 95 CI: 2.41–13.5, *p* < 0.001) but not IGAN, MCGN or MN. At 10 years, recurrence occurs in 8.7, 10.8, 13.1, and 13.4% of allografts for FSGS, IGAN, MCGN and MN respectively. In all GN, recurrence significantly reduced death censored graft survival at 5 and 10 years.

**Conclusions:**

GN recurrence occurs in a minority of patients at a significantly different rate for each GN. After a recurrence, there is no evidence for an increased risk of further recurrence in a subsequent graft except in FSGS.

## Background

Glomerulonephritis (GN) is a major cause of end stage renal failure (ESRF) [1]. It represents the primary cause of end stage renal disease (ESRF) for 25% of the dialysis population [1] and 45% of the transplant population (Table 1). For patients with GN requiring renal replacement therapy, kidney transplantation is associated with superior outcomes compared with dialysis [2]. However, recurrence of GN is reported in 6–20% [3–8] of renal allograft recipients depending on duration of follow-up [5], local protocol biopsy practice, and type of primary GN [9]. Furthermore, recurrence of GN has a negative impact on long term graft outcomes with recurrence being the third most common cause of allograft loss after chronic rejection and death [3, 4].

**Table 1:** Baseline demographics for 16,023 kidney transplant recipients recorded in the ANZDATA registry from 1985 to 2013

	IgA	MCGN	MN	FSGS	Other GN	Total GN	Non-GN	*P* value
Number (% of Total GN)	2393 (33.07%)	348 (4.81%)	309 (4.27%)	975 (13.47%)	3211 (44.38%)	7236	8787	
Age (years) Median (IQR)	45 (36–54)	40 (29–51)	49 (38–58)	43 (32–55)	44 (32–55)	44 (33–54)	47 (33–56)	< 0.001
Male gender	1815 (75.8%)	200 (57.5%)	235 (76.1%)	651 (66.8%)	1891 (58.9%)	4792 (66.2%)	4967 (56.5%)	< 0.001
Caucasian Ethnicity (%)	1889 (78.9%)	286 (82.1%)	274 (88.7%)	839 (86.1%)	2584 (80.5%)	5872 (81.1%)	7667 (87.2%)	< 0.001
Graft Number								< 0.001
Primary graft	2162 (90.35%)	288 (82.76%)	278 (89.97%)	861 (88.61%)	2886 (89.88%)	6475 (89.5%)	8096 (92.14%)	
Secondary	215 (8.98%)	55 (15.80%)	30 (9.71%)	98 (10.05%)	288 (8.97%)	686 (9.5%)	629 (7.16%)	
Subsequent	16 (0.67%)	5 (1.44%)	1 (0.32%)	16 (1.64%)	37 (1.15%)	75 (1%)	62 (0.71%)	
Donor Category								< 0.001
Deceased donor	1552 (64.9%)	260 (74.7%)	210 (68.0%)	671 (68.8%)	2263 (70.5%)	4956 (68.5%)	6201 (70.6%)	
Live related donor	557 (23.2%)	57 (16.4%)	71 (23.0%)	207 (21.2%)	684 (21.3%)	1576 (21.8%)	1686 (19.2%)	
Live unrelated donor	284 (11.9%)	31 (8.9%)	28 (9.1%)	97 (9.9%)	264 (8.2%)	704 (9.7%)	900 (10.2%)	
DM as comorbidity (% of Total GN)	73 (3.1%)	18 (5.2%)	15 (4.9%)	57 (5.8%)	103 (3.2%)	266 (3.7%)	1938 (22.1%)	< 0.001
Peak panel reactive antibodies (%) Median (IQR)	3 (0–14)	5 (0–29)	3 (0–16)	3 (0–21)	4 (0–25)	3 (0–20)	3 (0–16)	< 0.001
Total ischaemic time in hours (Median (IQR))	10 (3–15)	13 (4–19)	11 (3–16)	11 (3–16)	12 (4–17)	11 (3–16)	11 (4–16)	< 0.001
Zero HLA mismatches n(%)	179 (7.5%)	25 (7.2%)	21 (6.8%)	63 (6.5%)	245 (7.7%)	534 (7.4%)	597 (6.8%)	0.55

The risk of recurrence and its impact on outcomes are important questions for patients and clinicians in considering transplantation. It is unclear how GN recurrence in a first allograft impacts the risks of recurrence and graft survival in subsequent kidney transplants. To determine the consequences in subsequent kidney allografts we examined the incidence and timing of allograft loss due to biopsy-proven recurrence of GN from the Australia and New Zealand Dialysis and Transplant registry (ANZDATA), evaluating risk factors for allograft loss and the impact of GN recurrence in first kidney allografts on subsequent kidney transplants based on 28 years of transplant registry data.

## Methods

### Study population

Data were extracted from ANZDATA for all renal allografts transplanted for patients with a biopsy proven GN between 1985 and 2013 within Australia and New Zealand. Transplants included first, second, or subsequent transplants. In analyses of “Primary Glomerulonephritis” we included only patients with IgA Nephropathy (IGAN), Focal Segmental Glomerulosclerosis (FSGS), Membranous Nephropathy (MN) and Mesangiocapillary Glomerulonephritis (MCGN), excluding “Other GN”. All data is reported annually by individual units and the diagnosis of GN is determined by individual units.

### Outcomes

Primary end points were recurrence of GN and death censored allograft loss until December 2013. Recurrence of GN is reported by individual units with the date of the biopsy. Death censored allograft loss is defined by the need to commence renal replacement therapy by dialysis or repeat kidney transplantation.

### Statistics

All statistical analyses were carried out in R [10]. Baseline characteristics among groups were assessed using Pearson’s chi-square test and one way ANOVA. Significance of non-parametric data was assessed by Mann-Whitney U and categorical data by χ^2^. Survival analyses were performed in R, using the Surv function from the survival library [11] and the npsurv and survplot functions from the rms library [12]. Graphs are plotted with 95% confidence intervals. Cox proportional hazards ratios are calculated using the coxph function from the survival library, with testing of assumptions in cox.zph. The Cox model included donor category, age category, dialysis vintage, peak PRA, gender, recurrence, and total ischemic time. A time interaction variable was introduced for GN recurrence in the cox model for death censored graft survival.

## Results

A total of 16,023 kidney transplants were performed during the period 1985–2013 of which 7236 (45.16%) were attributed to biopsy-proven GN. As expected, the majority of transplants performed for GN within this period were primary allografts (*n* = 6475, 89.5%) followed by secondary (*n* = 686, 9.5%) and subsequent allografts (*n* = 75, 1%). Median follow up was 6.1 years (IQR 2.46–11.47). Baseline demographics are summarised in Table 1.

### Incidence and time to post-transplant glomerulonephritis recurrence

GN recurrence occurred in 511 (7.06%) of any kidney allografts in patients with GN within the follow-up period. In GN attributed to a primary GN (IGAN, FSGS, MN or MCGN) there were 424 recurrences from a total of 4025 transplants (10.5%). Overall, recurrence in either primary or secondary allograft was highest for MCGN (15.52%) followed by MN (12.30%) and lowest in Other GN (2.71%) (Table 2).

**Table 2:** Recurrence rates for glomerulonephritis in primary, secondary and subsequent kidney allografts (n of N(%))

	FSGS *n* (%) (*n* = 975)	IGAN *n* (%) (*n* = 2393)	MCGN *n* (%) (*n* = 348)	MN *n* (%) (*n* = 309)	Other GN *n* (%) (*n* = 3211)
Total	101 (10.4%)	231 (9.7%)	54 (15.5%)	38 (12.3%)	87 (2.7%)
Primary graft	79 of 861 (9.1)	210 of 2162 (9.7)	48 of 288 (16.6)	38 of 278 (13.6)	76 of 2886 (2.6)
Secondary graft	19 of 98 (19.3)	20 of 215 (9.3)	6 of 55 (10.9)	0 of 30 (0)	10 of 288 (3.4)
Subsequent grafts	3 of 16 (18.7)	1 of 16 (6.2)	0 of 5 (0)	0 of 1 (0)	1 of 37 (2.7)

Primary allograft recurrence was highest for MCGN (*n* = 48, 16.6%). In comparison to previous reports, FSGS was reported with the lowest recurrence rates (*n* = 101, 10.4%). For second allografts recurrence was greatest for FSGS (19.3%) followed by MCGN (10.9%) (Table 2).

**Fig. 1:**
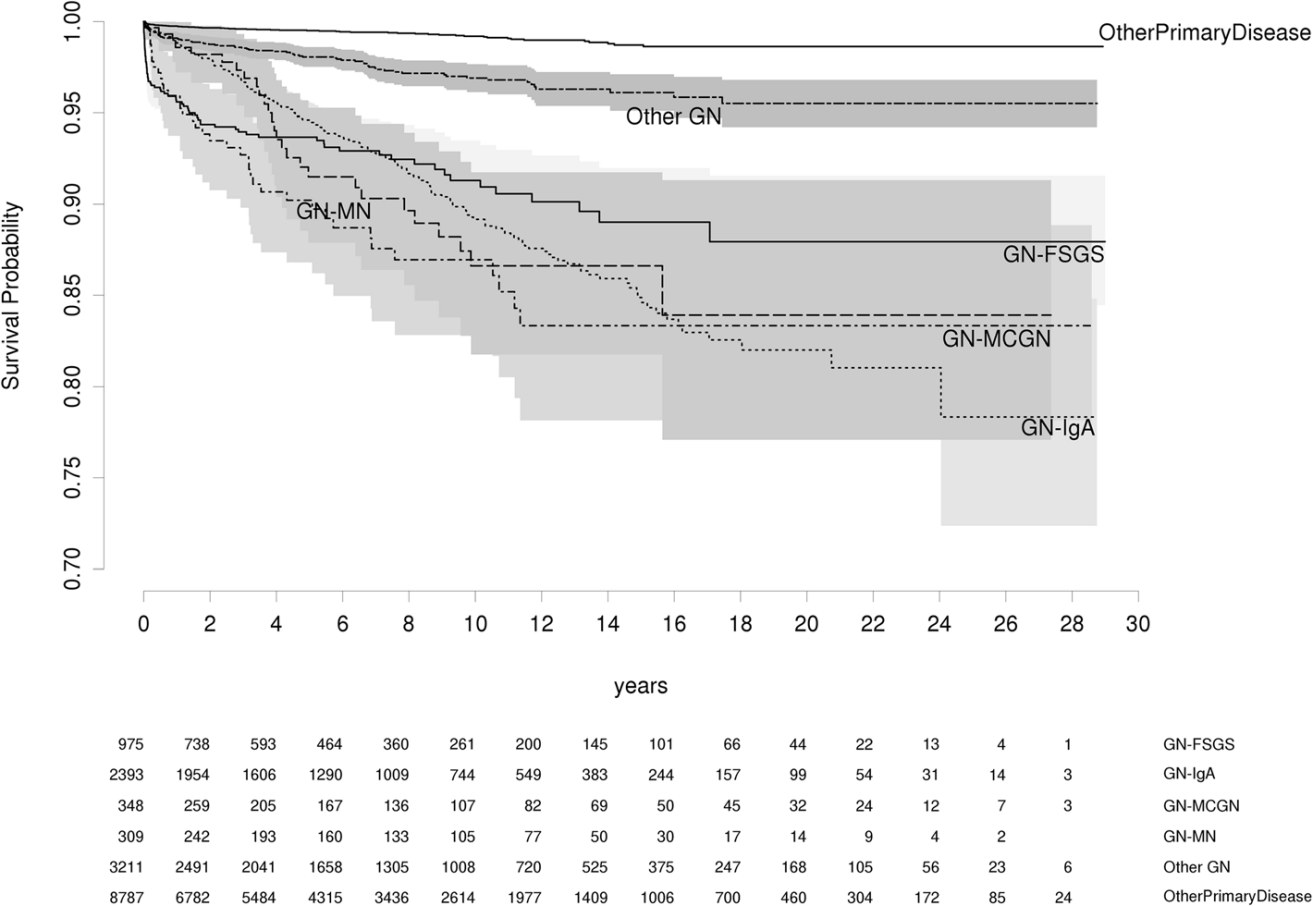
Recurrence free survival for each disease category

To determine whether time to recurrence differed by GN, we examined recurrence free survival in any allograft for each GN (Fig. 1.) When compared with IGAN, recurrence of FSGS and MCGN occurred with significantly shorter median times to recurrence (0.56 vs 4.63 years, FSGS vs IGAN, *p* < 0.0001, 1.87 vs 4.63 years, MCGN vs IGAN, *p* = 0.0003) (Table 3). Recurrence rates at 5 and 10 years are shown in Table 4.

**Table 3:** Number of patients with documented time to recurrence. Time to recurrence in Years

	Number with time to recurrence	Median (IQR) Years
FSGS	72 of 102	0.56 (0.05–2.91)
IGAN	201 of 231	4.63 (2.12–8.66)
MCGN	38 of 54	1.87 (0.52–4.88)
MN	27 of 38	3.93 (2.91–6.47)
Other	76 of 87	2.52 (0.39–6.66)

**Table 4:** Recurrence rates for Primary GN at 5 and 10 years post-transplant

	5 years	10 years
FSGS	6.3% (4.7–7.9)	8.7% (6.5–10.8)
IGAN	5.4% (4.3–6.5)	10.8% (9.2–12.5)
MCGN	9.8% (6.2–13.2)	13.1% (8.7–17.2)
MN	8.5% (4.7–12.1)	13.4% (8.3–18.2)
Other	1.9% (1.4–2.5%)	3.1% (2.3–3.9)

### Recurrence of FSGS in a primary graft increases risk of recurrence in a subsequent graft

We then examined the implication of GN recurrence in a first allograft for risk of recurrence in a subsequent transplant. We compared rates of recurrence in secondary grafts between patients with or without recurrence in their primary kidney allograft. FSGS has a significantly higher risk of recurrence in secondary grafts after a prior recurrence (RR 5.70, 95 CI: 2.41–13.5, *p* < 0.001) (Table 5). Recurrence in a primary allograft was not associated with increased risk of recurrence in subsequent kidney allografts in IGAN (RR: 1.24, 95 CI: 0.53–2.91, *p* = 0.62) or MCGN (RR: 1.79, 95 CI: 0.37–8.63 *p* = 0.5) (Table 5). Therefore, recurrence in primary kidney allografts is associated with increased risks of recurrence in secondary allografts for patients with FSGS, but not IGAN nor MCGN.

**Table 5:** Incidence of GN recurrence in secondary grafts analysed by outcome of primary grafts

Incidence of GN recurrence in secondary grafts			
	Primary Graft Outcome		
	Recurrence	Non-Recurrence	Risk Ratio of recurrence in secondary graft after recurrence in the first
FSGS	13 of 27 (48.2%)	6 of 71 (8.5%)	5.70 (2.41–13.5)
IGAN	8 of 75 (10.6%)	12 of 140 (8.6%)	1.24 (0.53–2.91)
MCGN	2 of 12 (16.6%)	4 of 43 (9.3%)	1.79 (0.37–8.63)
MN	0 of 8 (0%)	0 of 22 (0%)	NA

### GN recurrence reduces kidney allograft survival

**Fig. 2:**
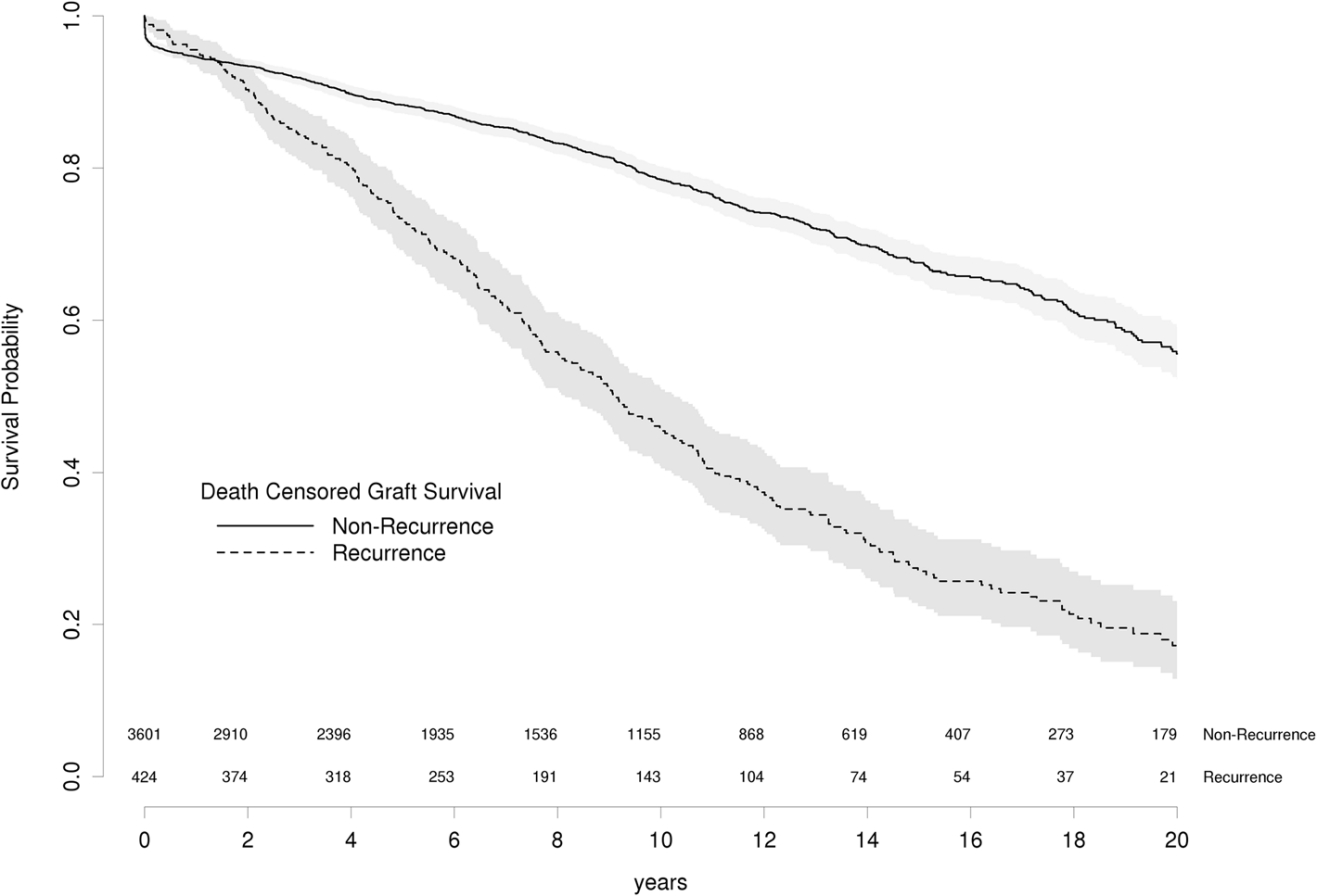
Death Censored Graft Survival in primary GN patients

**Fig. 3:**
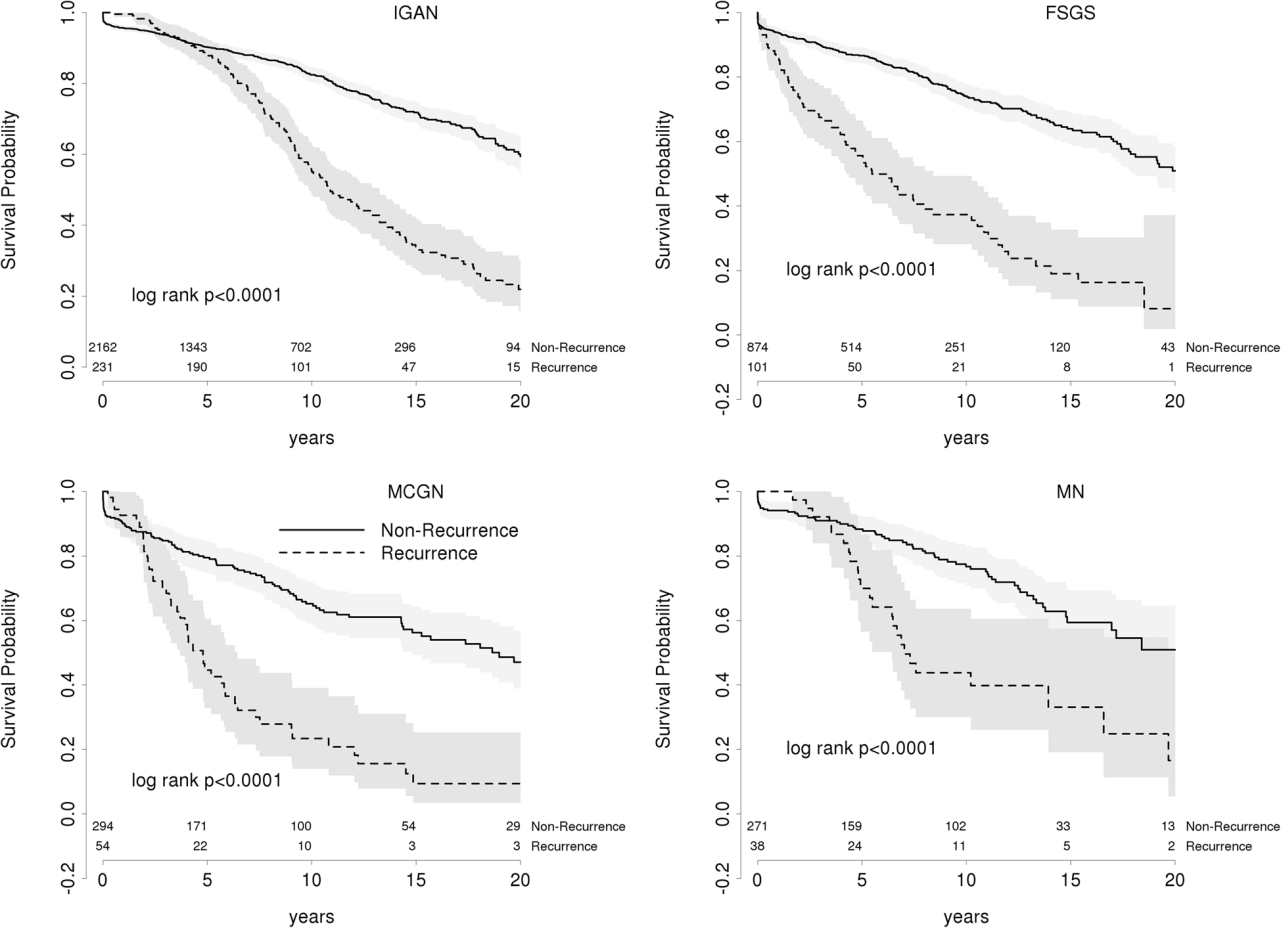
Death censored graft survival in each of the primary GNs

To estimate the effect of recurrence on allograft survival, we examined survival in kidney allografts with or without recurrence. As expected, death censored graft survival is significantly reduced in patients with recurrence of any GN (HR 3.1, 95CI: 2.38–3.97 *p* < 0.001) (Fig. 2) and for each primary GN (Fig. 3, Table 6). In all GN except FSGS, graft survival is superior for recurrent patients over the first twelve months. This most likely reflects ascertainment bias through selection against patients with early graft failure from early acute rejection or surgical complications. The earlier recurrence of FSGS in the post-operative period competes with the risks of these early causes of graft failure. Death censored graft survival at 5 and 10 years for recurrent and non-recurrent patients are presented in Table 6 with hazard ratios for each GN in Table 7.

**Table 6:** Death Censored Graft Survival at 5 and 10 years post transplant in patients with and without GN recurrence

	5 years		10 years	
	Recurrent	Non-recurrent	Recurrent	Non-recurrent
IGAN	88.3 (84.1–92.6)	90.2 (88.9–91.6)	55.1 (48.5–62.5)	82.4 (80.4–84.5)
FSGS	55.6 (46.5–66.5)	86.6 (84.3–89.1)	37.3 (28.2–49.4)	74.4 (70.5–77.9)
MCGN	44.6 (32.8–60.5)	79.5 (74.8–84.6)	23.4 (14–39)	65.2 (59.1–0.72)
MN	70 (56.6–86.6)	88.3 (84.2–92.5)	43.7 (30–63.7)	76.8 (70.8–83.2)

**Table 7:** Hazard ratios for death censored graft survival in patients with recurrent GN

GN	HR (95% CI)	*P* value
IgA	4.49 (2.70–7.47)	< 0.001
FSGS	1.83 (1.12–3.0)	0.02
MCGN	3.14 (2.16–4.57)	< 0.001
MN	2.20 (1.26–3.86)	0.03

**Fig. 4:**
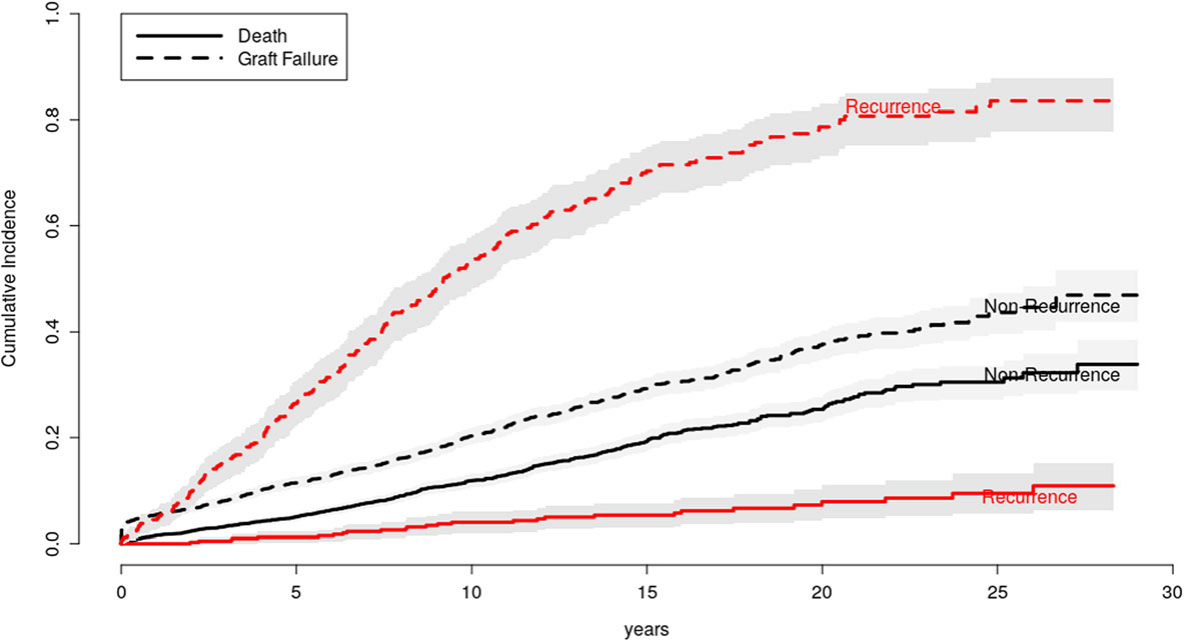
Competing risk analysis of death and graft failure. Patients with recurrent GN have a lower risk of death but a higher risk of graft failure

Whilst death censored graft survival is reduced in GN recurrence, the overall effect of recurrence is more complex. We performed a competing risk analysis of death and graft failure by GN recurrence, which showed the recurrent patients have a lower risk of death but a higher risk of graft failure (Fig. 4). However, adjusting for age, gender and the lower prevalence of diabetes accounted for the lower risk of death in patients with GN recurrence (data not shown).

### Transplant outcomes and graft loss

One thousand nine hundred fourteen primary GN patients lost their graft of whom 329 (17.19%) had a documented recurrence of GN. Causes of graft loss have been analysed in GN and non-GN patients (Table 8) and in patients with and without GN recurrence (Table 9). GN patients have similar causes of graft loss to non-GN patients excluding loss due to GN recurrence. This is balanced by a reduction in loss due to rejection and chronic allograft nephropathy. Patients with recurrence have a significantly higher risk of graft failure due to GN (57%) and a lower risk of loss due to rejection when compared with GN patients without recurrence.

**Table 8:** Causes of graft failure in patients with and without GN

Cause	GN Patients	Other Patients
ATN	0.50%	1.00%
BK	1.00%	0.80%
CAN	55.40%	59.50%
Cortical Necrosis	1.30%	2.10%
Donor Malignancy	0.50%	0.30%
Drugs	1.30%	1.20%
Embolus	0.30%	0.30%
GN	13.50%	1.80%
Haemorrhage	0.90%	1.20%
HUS	0.70%	1.20%
Infection	1.10%	1.30%
Malignancy	0.40%	0.60%
Non-compliance	3.30%	3.70%
Other	2.50%	3.30%
Rejection	10.90%	13.80%
Urological	0.30%	0.70%
Vascular	6.10%	7.10%

**Table 9:** Causes of graft failure in GN patients with and without recurrence

Cause	Non-Recurrence	Recurrence
ATN	0.80%	0.00%
BK	1.00%	0.30%
CAN	60.50%	30.30%
Cortical Necrosis	1.10%	0.30%
Donor Malignancy	0.40%	0.00%
Drugs	1.30%	0.80%
Embolus	0.30%	0.30%
GN	2.30%	55.00%
Haemorrhage	1.20%	0.00%
HUS	0.70%	3.30%
Infection	1.30%	0.00%
Malignancy	0.50%	0.50%
Non-compliance	3.80%	0.80%
Other	2.70%	5.40%
Rejection	14.20%	2.60%
Urological	0.60%	0.00%
Vascular	7.30%	0.50%

### Age, gender, donor status and dialysis vintage are predictors of GN recurrence

**Fig. 5:**
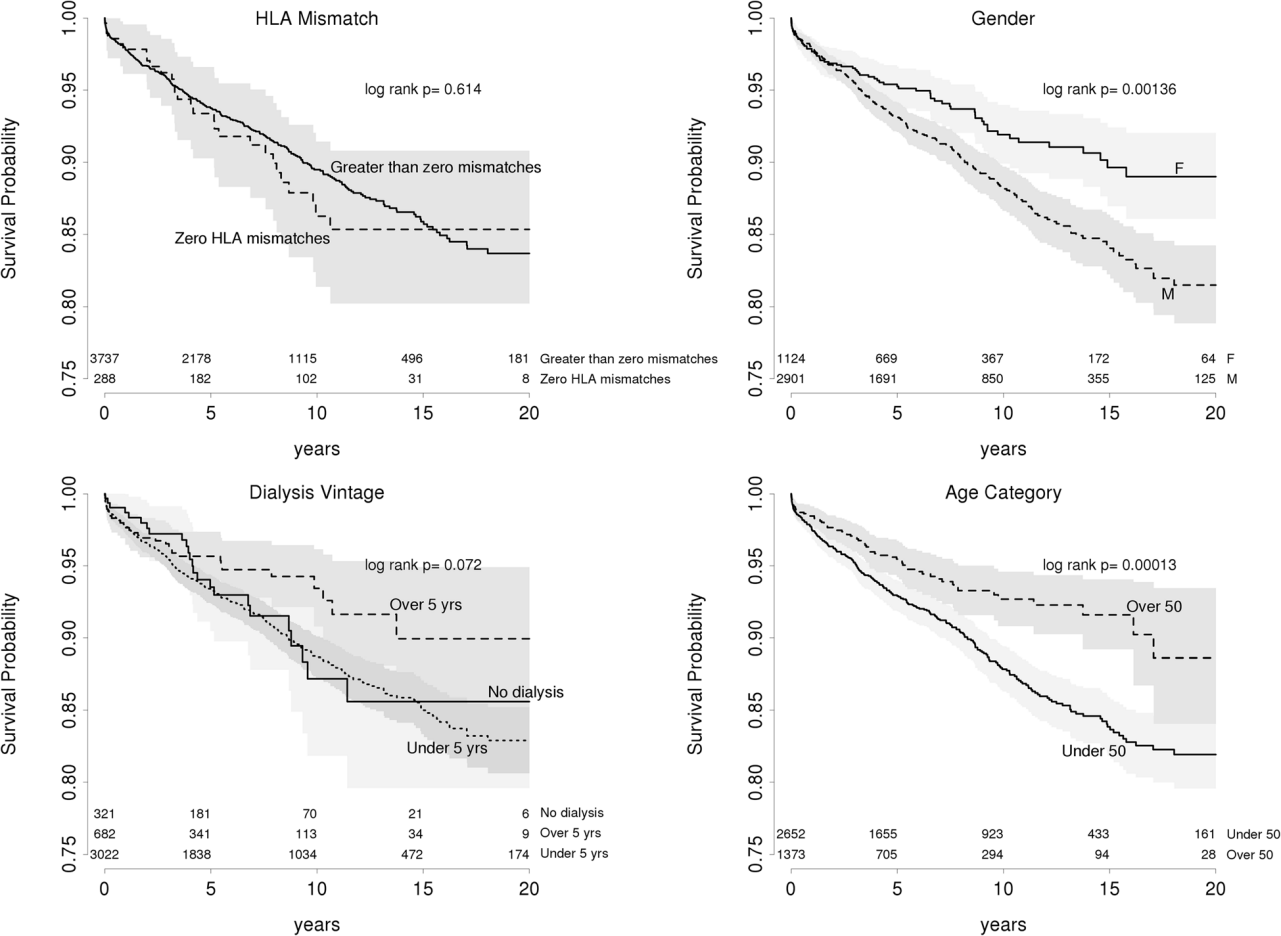
Recurrence free survival for the primary GNs. Age category and gender have a significant impact on risk of GN recurrence on univariate analysis. Dialysis for over 5 years prior to transplantation shows a trend towards reduced recurrence risk

We then examined variables influencing recurrence of GN. By univariate analysis, male gender, age below 50 years, duration on dialysis less than 5 years before transplant and peak panel reactive antibodies (PRA) were significantly associated with GN recurrence (Fig. 5). Multivariate analysis confirmed higher risk of recurrence evident for patients under 50 years of age (HR 1.59 95 CI: 1.21–2.09 *p* < 0.001), related kidney donation (HR 1.68, 95 CI: 1.31–2.15, *p* < 0.001) and male gender (HR 1.60, 95 CI: 1.23–2.08, *p* < 0.001) but dialysis vintage was not associated. Therefore, age below 50 years, male gender and a related donor are associated with increased risks of recurrence.

## Discussion

Our study reaffirms unique patterns of recurrence and disease progression for each of the four primary GNs. In general, recurrence rates are low occurring in 10–16% of patients over 12 years. Of the four primary GNs, FSGS has the more aggressive natural history. Patients with FSGS had significantly faster onset of recurrence than the other primary GNs. This study reinforces the risk of early and aggressive recurrence associated with FSGS and MCGN which has been reported elsewhere [4, 13–15].

FSGS was the only GN in which recurrence in a first transplant predicts greater risk in subsequent grafts, although the rate of recurrence in the primary allograft was lower than expected. This may reflect ascertainment or classification bias in the diagnosis of rapidly failing primary allografts. In IGAN and MCGN, recurrence in the primary allograft does not appear to predict the risk of disease recurrence in subsequent allografts. Previous reports by Ohmacht et al. suggested higher risks in IGAN for second grafts [16]. Freese reported 6 patients with IGAN in a transplant undergoing a second graft [17], wherein one patient developed recurrence. The largest series of recurrence for any GN reported so far came from Briggs with 23 of 48 patients (48%) developing graft failure due to recurrence in second grafts [13]. These were in all forms of GN including childhood FSGS and MCGN, which makes it difficult to interpret.

In examining variables that may contribute to recurrence, our study indicates that age and male gender are associated with greater risks of GN recurrence. Interestingly, living related donation was also significantly associated with increased risks of GN recurrence suggesting a genetic contribution to GN recurrence.

Other reports present conflicting observations of the effect of recurrence on allograft survival. Moroni et al. [18], Ponticelli [19] and Kim [20] reported high rates of GN recurrence but no effect of recurrence on allograft survival. These studies all suffered from relatively low study numbers. Moriyama studied 49 patients with IGAN who were transplanted. All patients had their allografts biopsied on implantation, 13 of them were shown to have latent IgA deposits already [21]. They reported a 26% IGAN recurrence rate at 5 years, higher than ours, but interestingly demonstrated that latent IgA deposition in the graft at donation was a significant risk for both GN recurrence and subsequent graft failure Conversely, Bumgardner reported recurrence of IGAN in 18 of 61 transplants with a mean follow-up time of 5 years with significant graft loss due to recurrence [22].

In this study, recurrence of GN was associated with a significant reduction in kidney allograft survival. Our survival analyses suggest improved graft survival in the first year of transplant for each primary GN except FSGS. This likely reflects ascertainment bias as patients with early graft failure from early acute rejection or surgical complications will therefore not be diagnosed with earlier recurrence of FSGS in the post-operative period.

Previous registry studies have suggested that graft half-life is less than 5 years with GN recurrence with a relative risk of 1.9 calculated for graft loss [23]. In this study, grafts with GN recurrence have a half life of approximately 10 years and a Hazard Ratio of 3.1 for death censored graft failure compared to non-recurrent grafts. MCGN, MN and FSGS tend to have shorter graft half lives. We are in agreement with a more recent single centre study though their analysis did not show GN recurrence as a risk for death censored graft failure [18]. Moroni analysed 190 IGAN transplants and compared them with 380 non-diabetic controls, suggesting that IGAN patients had worse survival than the controls but recurrence of disease had little impact on graft survival for the first 10 years [24]. In chinese patients with IGAN, Choy reported a 20% recurrence rate at 10 years but with little impact on graft survival [25]. The estimates in our study are based on a much larger cohort and are likely to be more robust.

The strength of our analysis lies in the large database with a well-characterised study population and complete dataset with up to 28 years follow-up. There are limitations inherent in Registry analysis in terms of heterogeneity of clinical practice across contributing units. Over the period of data collection, disease definition has changed. FSGS and MCGN are now regarded as multiple disease entities. It is not possible to retrospectively reclassify these diseases and it is important to keep this in mind in interpreting our data. Also the diagnosis of recurrent GN was conducted by each hospital. They was no independent examination of the diagnosis possible. Also the reason for the kidney biopsy was not included in this analysis and indeed was not available for this dataset. Despite these limitations, this study represents the most complete and well-populated registry analysis to date.

The increasing use of protocol biopsies is likely to increase reported risk of GN recurrence but should be viewed by clinicians with caution. This aggressive approach to transplant followup has yet to be shown to alter hard outcomes and, in the setting of recurrent GN, may be potentially misleading. The interpretation of immunoglobulin deposits in a donor kidney in the absence of clinical disease is far from clear. As demonstrated in the implantation biopsies of donor kidneys [21], the presence of IgA deposits in a kidney does not necessarily imply disease recurrence in the recipient and may instead be a marker of the donor’s genetic predisposition to renal disease. At a minimum, the research community needs to establish that protocol biopsy data is clinically meaningful in terms of recurrent glomerulonephritis.

## Conclusions

Primary GN recurs in 10–16% of renal allografts over a 15 year period. FSGS and MCGN tend to recur earlier than IGAN. After recurrence, graft survival is significantly reduced. Transplants performed after a previous recurrence have similar risks of recurrence, except in the case of FSGS, which is more likely to recur. Patients and clinicians need to be aware of the risks of recurrence and its consequences.

## Abbreviations

ANZDATA: Australia and New Zealand Dialysis and Transplant registry
CI: Confidence Interval
DCGS: Death Censored Graft Survival
ESRF: End Stage Renal Failure
FSGS: Focal and Segmental Glomerulosclerosis
GN: Glomerulonephritis
HR: Hazard Ratio
IGAN: IgA Nephropathy
MCGN: Mesangiocapillary Glomerulonephritis
MN: Membranous Nephropathy
